# Mode of inhibition of HIV-1 Integrase by a C-terminal domain-specific monoclonal antibody*

**DOI:** 10.1186/1742-4690-3-34

**Published:** 2006-06-21

**Authors:** Joseph Ramcharan, Diana M Colleluori, George Merkel, Mark D Andrake, Anna Marie Skalka

**Affiliations:** 1 The Institute for Cancer Research, Fox Chase Cancer Center, 333 Cottman Avenue, Philadelphia, PA 19111, USA; 2 Locus Pharmaceuticals, Inc., 4 Valley Square, 512 E. Township Line Road, Blue Bell, PA 19422, USA; 3 App Tec, Inc., 4751 League Island Blvd., Philadelphia, PA 19112, USA

## Abstract

**Background:**

To further our understanding of the structure and function of HIV-1 integrase (IN) we developed and characterized a library of monoclonal antibodies (mAbs) directed against this protein. One of these antibodies, mAb33, which is specific for the C-terminal domain, was found to inhibit HIV-1 IN processing activity *in vitro*; a corresponding Fv fragment was able to inhibit HIV-1 integration *in vivo*. Our subsequent studies, using heteronuclear nuclear magnetic resonance spectroscopy, identified six solvent accessible residues on the surface of the C-terminal domain that were immobilized upon binding of the antibody, which were proposed to comprise the epitope. Here we test this hypothesis by measuring the affinity of mAb33 to HIV-1 proteins that contain Ala substitutions in each of these positions. To gain additional insight into the mode of inhibition we also measured the DNA binding capacity and enzymatic activities of the Ala substituted proteins.

**Results:**

We found that Ala substitution of any one of five of the putative epitope residues, F223, R224, Y226, I267, and I268, caused a decrease in the affinity of the mAb33 for HIV-1 IN, confirming the prediction from NMR data. Although IN derivatives with Ala substitutions in or near the mAb33 epitope exhibited decreased enzymatic activity, none of the epitope substitutions compromised DNA binding to full length HIV-1 IN, as measured by surface plasmon resonance spectroscopy. Two of these derivatives, IN (I276A) and IN (I267A/I268A), exhibited both increased DNA binding affinity and uncharacteristic dissociation kinetics; these proteins also exhibited non-specific nuclease activity. Results from these investigations are discussed in the context of current models for how the C-terminal domain interacts with substrate DNA.

**Conclusion:**

It is unlikely that inhibition of HIV-1 IN activity by mAb33 is caused by direct interaction with residues that are essential for substrate binding. Rather our findings are most consistent with a model whereby mAb33 binding distorts or constrains the structure of the C-terminal domain and/or blocks substrate binding indirectly. The DNA binding properties and non-specific nuclease activity of the I267A derivatives suggest that the C-terminal domain of IN normally plays an important role in aligning the viral DNA end for proper processing.

## Background

HIV-1 Integrase (IN)^1 ^is a 32-kDa viral protein that is required for the insertion of viral DNA into the chromosome of the host cell, an essential step in the life cycle of retroviruses [1-3]. Because of its critical role, IN is as an attractive target for the design and screening of novel drugs against AIDS [4]. IN catalyzes the first two steps of the three-step DNA integration process. In the first step called processing, IN nicks the 3'-ends of the viral DNA, releasing two nucleotides from each of the 3'-OH ends. In the second step, IN catalyzes a concerted cleavage-ligation reaction in which both 3'-processed viral DNA ends are joined to the host-cell chromosomal DNA. The IN protein is composed of three distinct domains: an N-terminal domain (NTD); a catalytic core domain (CCD); and a C-terminal domain (CTD) [5-7]. The NTD (residues 1–50) comprises a three-helix bundle containing a conserved HHCC motif, which chelates one Zn^2+ ^[8,9]. This region was shown to promote IN protein oligomerization [10]. The CCD (residues 50–212) contains a conserved D,D(35)E motif, which comprises the active site of IN and which binds at least one divalent metal cofactor, Mg^2+ ^or Mn^2+^, required for enzymatic activity [11-14]. The CTD (residues 213–288) is important for binding of viral and possibly host DNA [15-18]. The isolated CTD adopts an SH3 fold and forms a dimer in solution [19,20]. However, it should be noted that only the CCD displays the same dimer interface in all crystal structures determined to date. There is considerable variation among CTD interfaces in crystal structures of two-domain derivates of IN that include the CTD [21]; some of these interfaces are seen only across symmetry-related molecules in the crystals.

Various lines of evidence indicate that HIV-1 IN undergoes a conformational change upon addition of the Mg^2+ ^or Mn^2+ ^cofactor, and that this change promotes preferential and stable binding to its viral DNAsubstrate [22-24]. We have developed a library of monoclonal antibodies to HIV-1 IN one of which, mAb33, is specific for the CTD, but binds tightly only to the apo-enzyme. Binding of mAb33 prevents the metal-induced conformational change and inhibits the enzymatic activity of IN. If metal and substrate DNA are added before the antibody, inhibition of IN activity is greatly reduced [25]. These observations are consistent with a model in which the mAb33 epitope becomes inaccessible in the ternary IN•Metal•DNA conformation. However, we have also shown that the Fab fragment of mAb33 blocks DNA binding to the isolated CTD [26]. Therefore, it was conceivable that this antibody also blocks DNA binding to full length HIV-1 IN either by competing for the same or overlapping binding sites, or by distorting or constraining the structure of the CTD. Because intracellular expression of an scFv fragment derived from mAb33 blocks HIV-1 replication [27], its epitope must be accessible in infected cells and could therefore be a valuable target for developing inhibitors for AIDS therapy.

**Figure 1:**
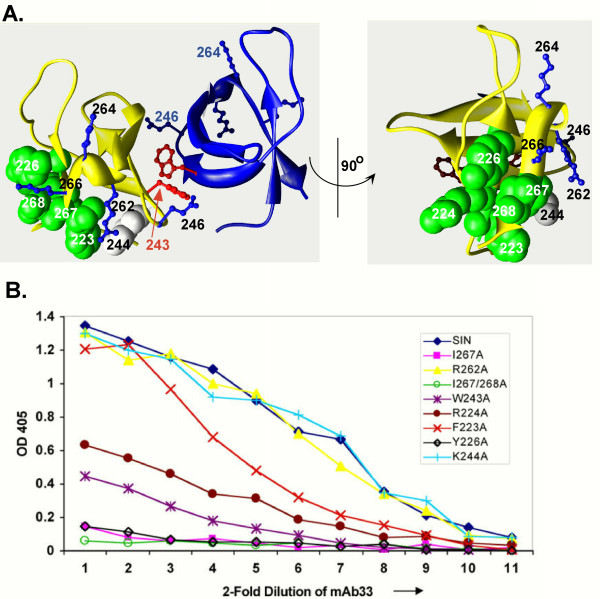
**Ala substitution of residues in the NMR-determined epitope of mAb33 decrease antibody binding**. (A) Model of the HIV-1 CTD dimer. *Left panel*: The NMR-determined SH3-fold structure of the CTD is displayed with one subunit colored in yellow and the other in blue. DNA binding residues E246, K264, K266, and R262 are shown in ball-and-stick representation and labeled with black lettering. Residues of the putative mAb33 epitope, as determined by NMR techniques [28], are displayed in green space filling representation. K244 is shown in grey, as it is unlikely to be a critical component of the mAb33 epitope (see Panel B). The long arrow points to residue W243 at the interface between subunits in this NMR dimer of the CTD which forms a "saddle" with both K264 residues extending into the cleft proposed to bind DNA [19]. *Right panel*: An orthogonal view rotated 90 degrees about the displayed axis. This view shows residues known to be involved in DNA binding on one face of the subunit and adjacent to the mAb33 epitope. (B) ELISA data for CTD substituted HIV-1 sIN proteins and their interactions with mAb33. A high binding microtiter plate was coated with 50 ng of antigen (sIN or one of the Ala substituted sIN proteins) and incubated overnight at 4°C. The plates were then blocked with bovine serum albumin, washed, and incubated with the mAb33 1° antibody which was serially diluted (2-fold) from a starting concentration of 250 ng per well. A standard ELISA protocol was then followed using an alkaline phosphatase conjugated 2° antibody against the kappa chain. The relative binding efficiency of mAb33 to the IN proteins was determined by measuring the absorbance at 405 nm.

We reported previously that binding of mAb33 restricts the mobility of six contiguous, solvent accessible residues in the isolated CTD as determined by nuclear magnetic resonance (NMR) spectroscopy. We proposed that these residues, F223, R224, Y226, K244, I267, and I268 (Figure 1A), are included in the epitope for this antibody. Here we test this hypothesis by measuring the affinity of mAb33 to HIV-1 IN proteins that contain Ala substitutions in each of these positions. To gain additional insight into the mode of inhibition by mAb33 and the role of the CTD in HIV-1 IN activity, we also measured the DNA binding capacity and enzymatic activities of these Ala substituted proteins.

## Results

### Expression, purification, and characterization of HIV-1 IN proteins

To facilitate our SPR studies it was advantageous to increase the solubility of IN. The mutations that were introduced for this purpose encoded proteins with the following additional substitutions: 3CS/F185H, 3CS/F185K, 3CS/F185H/W131D/F139D, and 3CS/F185K/W131D/F139D. These changes also helped to reduce non-specific association of the IN proteins with the surface of the biosensor chip. These soluble derivatives were expressed and purified as described in Materials and Methods. The best yield of purified soluble protein was obtained with the 3CS/F185H/W131D/F139D derivative: approximately 40 mg per liter of culture, after cleavage of the 6XHis tag and the final purification step of Heparin column chromatography. Although all of the substituted IN proteins displayed similar -2 processing activity and mAb33 binding affinity (data not shown), we elected to utilize the 3CS/F185H/W131D/F139D-encoding construct for further mutagenesis studies because this protein exhibited the highest solubility. This hexa-substituted, soluble form of the HIV-1 IN protein was designated soluble IN (sIN). Several of the substitutions introduced into the CTD of sIN resulted in derivatives with decreased solubility as well as lower affinity for the Heparin column compared to the sIN protein. For this reason, the 6XHis tag was left on sIN and the CTD substituted sIN proteins for the studies presented here. The yield of purified His-sIN from a 1L expression culture was 80 mg. There were no significant differences observed between sIN and His-sIN in their ability to bind mAb33 or DNA, and the proteins had comparable catalytic activities (data not shown).

### Rationale for amino acid substitutions

The binding of mAb33 was previously reported to reduce mobility of contiguous residues on the surface of the β-strands of the isolated CTD [28]. It was proposed that these residues, F223, R224, and Y226 on β_1_, K244 on β_2_, and I267 and I268 on β_5 _(Figure 1A) comprise the mAb33 epitope. To determine if these amino acids are important for mAb33 recognition, alanine-scanning mutagenesis was used. Residues R262 and W243, which are adjacent to the putative antibody binding site and could be affected by antibody binding, were also changed to produce Ala substituted derivatives. The R262 residue has been implicated in DNA binding [29] and modeling studies suggested that the close proximity of R262 to the mAb33 binding site might account for decrease binding of IN to DNA in the presence of the corresponding Fab fragment, Fab33 [28]. W243 has been implicated in CTD dimer interactions and is adjacent to the putative epitope residue, K244. Comparison of residues protected from protease digestion by Fab33 and another CTD-specific monoclonal, Fab32, revealed that Fab33 interacts with I267 and I268, whereas Fab32, which has no effect on DNA binding, does not [30,31]. We therefore chose to analyze these residues in greater detail by generating both of the single substitutions, I267A and I268A, as well as the doubly substituted sIN protein, I267A/I268A.

### Substitution of putative epitope residues affects antibody binding

The relative binding affinities of mAb33 to sIN and the Ala substituted sIN proteins were assessed by the enzyme-linked immunosorbent assay (ELISA). Alanine substitution of five of the six residues comprising the putative mAb33 epitope resulted in a significant reduction in the binding affinity of mAb33 (Figure 1B). Interestingly, the greatest reductions were seen with Ala substitution of the hydrophobic residues, Y226A, I267A, and I268A. The R224A, and F223A sIN proteins were less seriously compromised for mAb33 binding. The K244A derivative was largely unaffected in mAb33 binding, consistent with the variable results observed in our previous NMR studies [28]. This residue is unlikely therefore to play a direct role in antibody binding. The non-epitope substitution R262A derivative binds mAb33 as well as the parental protein sIN, as expected from previous NMR studies [28], while W243A, which may be involved in dimer interactions [21], is compromised for antibody binding.

As a control, sIN and the Ala substituted sIN proteins were also analyzed for their abilities to bind NTD-specific mAb17 and CCD-specific mAb4 [31,32]. No significant differences were observed in the abilities of the substituted derivatives to interact with these monoclonal antibodies as compared with sIN (data not shown). These data indicate that the NTDs and CCDs of the derivative proteins are not grossly disordered.

### Effect of substitutions in epitope and non-epitope residues on IN activity

**Figure 2:**
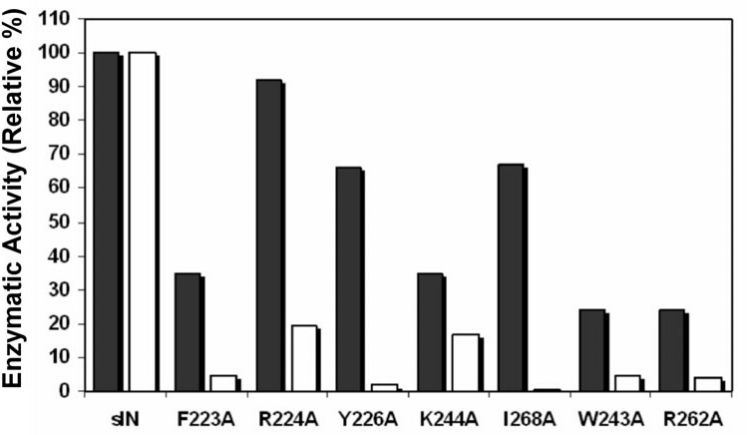
**Enzymatic Activity of HIV sIN and the CTD substituted sIN proteins**. Processing (solid bars) and joining (open bars) activities of the sIN derivatives are shown relative to that of sIN, whose activity is set at 100%. Assay conditions are described in Materials and Methods.

The effects of the CTD substitutions on enzymatic function were determined by assaying for the processing and joining activities using model duplex oligonucleotides that represent the viral U5 DNA end [2]. Results from these experiments, summarized in Figure 2, show that the proteins with substitutions in the mAb33 epitope retained 35% or more of the processing activity observed with the parental sIN. Proteins with substitutions in the non-epitope residues W243A and R262A were more defective. Only the R224A and K244A derivatives retained significant joining activity compared to the sIN, indicating that the epitope residues are important for protein function.

**Figure 3:**
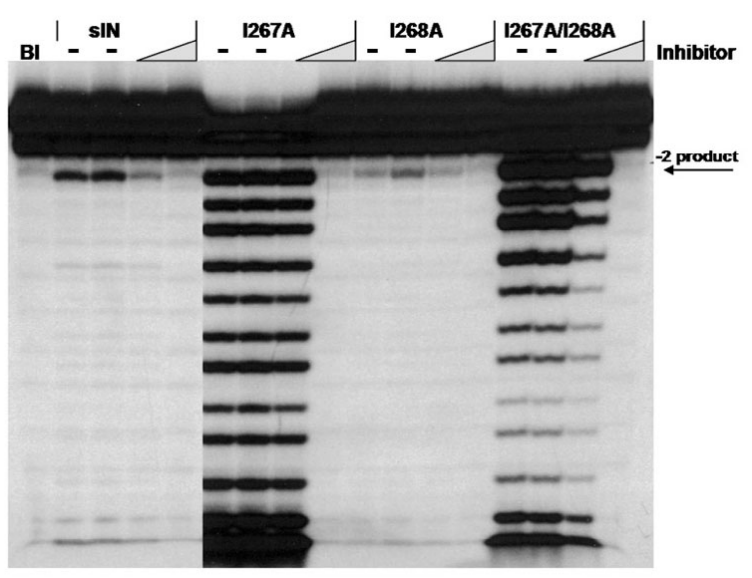
**Non-specific nuclease activity of I267A sIN derivatives**. Reaction conditions are described in Materials and Methods. Bl represents a blank reaction in which the enzyme was omitted. (-) indicates duplicate reactions in which the Merck HIV-1 inhibitor L-708,906 was absent. Either 10 or 100 μM of the L-708,906 inhibitor was added as indicated. These concentrations of inhibitor are expected to affect the processing as well as the joining activity of wild type HIV-1 IN [33].

The data in Figure 3 show that the processing products obtained with the I267A derivative were strikingly different from the parental sIN. This IN protein appears to have lost specificity for the -2 position and behaves instead as a highly active, non-specific nuclease. The I267A/I268A derivative is also less specific, but its nuclease activity appeared to diminish with distance from the 3'-end. As these activities were blocked by addition of an HIV-1 IN inhibitor [33], as was the sIN control, they are not caused by nuclease contamination of the derivative proteins. These results indicate that determinants in the CTD affect the specificity of catalysis by the CCD.

### Effect of substitutions in epitope and non-epitope residues on DNA binding

Surface plasmon resonance spectroscopy was used to study the interactions of sIN and derivative proteins with model viral DNA substrates that represent a blunt U5 LTR end, a processed U5 LTR end, and host target DNA. It was reported that divalent cations, such as Mg^2+^, Mn^2+^, and Ca^2+^, can stimulate the binding of HIV-1 IN to model viral DNA substrates [22]. Divalent cations have been shown to induce a conformational change in the enzyme [25], which might allow it to recognize and bind to viral DNA ends preferentially. To determine if the substitutions in sIN affected the metal-induced stimulation and binding kinetics of the enzyme, we examined the effect of Mg^2+ ^on the association of these proteins with DNA. Our results with the parental sIN protein (Table 1) are consistent with those previously published by Yi *et al*. [28]; sIN shows a 2-fold increase in binding affinity for the unprocessed viral DNA in the presence of Mg^2+^, indicating that the solubility-enhancing substitutions do not alter the conformational change associated with viral DNA binding and/or recognition.

**Table 1:** The effect of metal ions on the kinetic parameters of HIV-1 sIN binding to DNA substrates

	**U5-End**			**U5-End Cut**			**Target**		
	
	*k*_on _10^5 ^M^-1 ^s^-1^	*k*_off _10^-3 ^s^-1^	***K*_d _(nM)**	*k*_on _10^5 ^M^-1 ^s^-1^	*k*_off _10^-3 ^s^-1^	***K*_d _(nM)**	*k*_on _10^5 ^M^-1 ^s^-1^	*k*_off _10^-3 ^s^-1^	***K*_d _(nM)**
sIN	1.7 ± 0.1	1.0 ± 0.1	**5.9 ± 0.7**	1.1 ± 0.1	1.3 ± 0.1	**11.8 ± 1.4**	1.0 ± 0.1	1.6 ± 0.2	**16.0 ± 2.6**
sIN no Mg	1.9 ± 0.2	2.1 ± 0.1	**11.1 ± 1.3**	2.7 ± 0.2	2.0 ± 0.1	**7.4 ± 0.7**	3.5 ± 0.2	2.1 ± 0.1	**6.0 ± 0.7**

The model U5 end biotin-labeled substrate (see Experimental Procedures) was immobilized on a SA chip. BIAcore data were collected by injecting different concentrations of sIN, ranging from 50 to 300 nM dimers, at a flow rate of 30 μL/min. No Mg^2+ ^ions were achieved by eliminating MgCl_2 _from the running buffer and including 0.5 mM EDTA in the running as well as the dilution buffer. The dissociation constant was calculated from the ratio of the average of the *off *and the apparent *on *rates. All injections were done at 25°C.

DNA binding data for the sIN derivative proteins are presented in Table 2. The results show that most proteins exhibit a modest preference for the blunt viral DNA end, compared with processed viral DNA or target DNA. The *K*_d _values for proteins containing F223A, R224A, Y226A, or K244A substitutions are comparable to that of the parental sIN with respect to all the model DNA substrates. These data indicate that the substituted residues do not play a significant role in DNA binding. Residue W243 which is not part of the mAb33 epitope but lies adjacent to it, shows about a 2-fold decrease in binding affinity compared with sIN. W243 is believed to play a role in a CTD dimer interaction that may be compromised in the W243A derivative [21]. Kinetic parameters could not be determined for the R262 protein because binding to the DNA sensor chip was barely detectable, as expected [29]. This result indicates that even though DNA interactions may occur with other domains in the protein, they are not tight enough to produce a measurable SPR signal with this protein. From this control we can conclude that the data in Table 2 comprises a valid readout for the contribution of CTD residues to interaction with DNA.

**Table 2:** Kinetic parameters for HIV-1 sIN substituted proteins binding to DNA substrates

	**U5-End**			**U5-End Cut**			**Target**		
	
	*k*_on _10^5 ^M^-1 ^s^-1^	*k*_off _10^-3 ^s^-1^	***K*_d _(nM)**	*k*_on _10^5 ^M^-1 ^s^-1^	*k*_off _10^-3 ^s^-1^	***K*_d _(nM)**	*k*_on _10^5 ^M^-1 ^s^-1^	*k*_off _10^-3 ^s^-1^	***K*_d _(nM)**
sIN	1.7 ± 0.1	1.0 ± 0.1	**5.9 ± 0.7**	1.1 ± 0.1	1.3 ± 0.1	**11.8 ± 1.4**	1.0 ± 0.1	1.6 ± 0.2	**16.0 ± 2.6**
F223A	2.1 ± 0.2	1.1 ± 0.1	**5.2 ± 0.7**	1.1 ± 0.2	1.6 ± 0.2	**14.5 ± 3.2**	0.8 ± 0.1	2.5 ± 0.2	**31.3 ± 4.7**
R224A	1.3 ± 0.2	0.9 ± 0.1	**6.9 ± 1.3**	1.1 ± 0.2	1.2 ± 0.2	**10.9 ± 2.7**	0.9 ± 0.2	1.5 ± 0.2	**16.7 ± 2.3**
Y226A	1.2 ± 0.2	1.0 ± 0.1	**8.3 ± 1.6**	0.8 ± 0.1	0.6 ± 0.1	**7.5 ± 1.6**	0.6 ± 0.1	0.7 ± 0.1	**11.7 ± 2.6**
I267A	0.9 ± 0.1	0.2 ± 0.1	**2.2 ± 1.1**	1.0 ± 0.1	0.3 ± 0.1	**3.0 ± 1.0**	0.8 ± 0.1	0.3 ± 0.1	**3.8 ± 1.6**
I267A/I268A	0.8 ± 0.1	0.2 ± 0.1	**2.5 ± 1.3**	0.9 ± 0.1	0.3 ± 0.1	**3.3 ± 1.2**	0.7 ± 0.1	0.2 ± 0.1	**2.9 ± 1.5**
K244A	1.9 ± 0.2	1.2 ± 0.2	**6.3 ± 1.2**	0.9 ± 0.1	1.0 ± 0.2	**11.1 ± 2.5**	0.9 ± 0.1	1.2 ± 0.2	**13.3 ± 2.7**
W243A	0.5 ± 0.1	0.7 ± 0.1	**14.0 ± 3.4**	0.8 ± 0.2	0.9 ± 0.1	**11.3 ± 0.3**	0.9 ± 0.1	1.4 ± 0.1	**15.6 ± 2.1**
R262A	-	-	ND	-	-	ND	-	-	ND

Three different model biotin-labeled substrates were immobilized on the surface of a SA chip as described in Experimental Procedures. BIAcore data were collected by injecting different concentrations of sIN, ranging from 50 to 300 nM dimers, at a flow rate of 30 μL/min. All data were collected using buffer containing 5 mM MgCl_2_. The dissociation constant was calculated from the ratio of the average of the *off *and the apparent *on *rates. All injections were done at 25°C. ND, kinetic parameters could not be determined due to low binding efficiency.

The most significant change was observed with the I267A and I267A/I268A substituted proteins, which showed more than a 2-fold increase in binding affinity compared with sIN. This difference results mainly from a decrease in the *off *rates. The additional I268A substitution did not appear to alter the binding affinity of I267A. Furthermore, unlike sIN and the other derivatives, I267A and I267A/I268A have very similar *K*_d _values for model viral and host DNA. Wild type IN and sIN both display a characteristic biphasic IN-DNA dissociation which is detected by SPR as a fast phase followed by a much slower dissociation. The dissociation kinetics of the I267A and I267A/I268A derivatives with all three model DNA substrates were altered compared to sIN; the fast dissociation was absent and only the slow dissociation kinetics were observed (data not shown).

## Discussion

We have shown that mAb33, which targets the CTD of HIV-1 IN, is an effective inhibitor of the enzyme *in vitro *[27,32,34,35], and that intracellular expression of the cognate Fv fragment blocks HIV-1 replication (27). Understanding the relationship between the structure and function of HIV-1 IN is important for the development of potent drugs that target this enzyme. Most small molecule inhibitors developed to date target the active site in the catalytic core domain; the CTD has been overlooked as a target for drug design. Because knowledge of how binding of mAb33 to its epitope inhibits IN activity may reveal a valuable drug target, we have examined this interaction in some detail. Our previous studies have uncovered an unexpectedly complex mechanism. We found that mAb33 binding occurs only in the absence of the metal cofactor and such binding prevents a conformational change in the full-length protein that normally occurs upon cofactor binding and which is required for enzyme activity [34, 35]. Subsequent studies with the Fab fragment of mAb33 and the isolated CTD showed that binding rendered this domain resistant to proteolysis and also blocked DNA binding.

We have considered three, non-exclusive hypotheses for the mechanism of inhibition of full length HIV-1 IN by mAb33: 1) mAb33 binding blocks DNA binding, either because the binding sites overlap or the binding pocket is distorted, 2) mAb33 binds to residues whose availability are critical for IN function, and 3) mAb33 binding has detrimental long-range or conformational effects. Our previous NMR studies, supported by results from the ELISA assays summarized in Figure 1B, have identified five key components of the epitope for this antibody. Such identification and analysis of Ala substituted derivatives have allowed us to test some of the predictions of these hypotheses.

Data summarized in Table 2 show that binding of DNA by the full-length IN protein is not reduced by substituting any one of the residues in the mAb33 epitope with Ala. These results show that none of these residues is absolutely required for substrate binding. It is unlikely, therefore, that there is an overlap of binding sites for substrate with mAb33, as proposed in hypothesis 1. It remains possible, however, that mAb33 hinders DNA access to nearby residues that are essential for substrate binding. The mAb33 epitope is in close proximity to amino acids E246, R262, K264, and K266, which were shown by mutagenesis and various DNA binding assays to be important for substrate binding [18,20,29,36,37]. Unfortunately, the importance of this proximity is difficult to evaluate because the manner in which DNA interacts with the CTD is not yet known.

It was first suggested that DNA might bind in a "saddle" between two CTDs that form a dimer in the NMR-determined structure of this domain (see Figure 1A, left) [19,20]. However, as such a saddle is not observed in the two-domain crystal structure of HIV IN (50–288) its significance to DNA binding is unclear [21]. Substrate DNA has also been proposed to bind to a different face of the CTD, where several known DNA binding residues cluster (see Figure 1A, right) [38,39]. Modeling studies by Zhu *et al*. [39] have identified residues in the CTD that might be involved in DNA binding to this region. Of these, residues K244 and I267 are predicted to form hydrogen bonds with the substrate DNA molecule in their model. Because we show that Ala substitution of either of these residues has no effect on DNA binding (Table 2), such hydrogen bonding cannot be essential for this function. Other recent studies have mapped the binding site for an IN inhibitor, pyridoxal 5' phosphate (PLP), to a region of the CTD close to that of the mAb33 epitope. These studies show that K244 is in contact with the PLP inhibitor, which is active in the low micromolar range [40,41]. However, as already noted, because our data (Table 2) show that Ala substitution of K244 does not reduce DNA binding, we suggest that PLP may inhibit HIV-1 IN by affecting the conformation or flexibility of the DNA binding pocket, rather than competing for the DNA binding site. Taken together, results from our analyses of the Ala derivatives fail to provide evidence that mAb33 inhibits the activity of full-length IN by competing directly for critical DNA binding residues, but leaves open the possibility that DNA binding could be blocked indirectly.

In the absence of knowledge of the exact spatial organization of CTDs within an active IN-DNA complex we cannot exclude the possibility that some of our Ala substitutions disrupt important domain interactions, and that this contributes to the decreases in enzymatic activity observed with these derivatives (Figure 2). Furthermore, although binding of the antibody to epitope residues might not be expected to produce the same phenotype as substituting epitope residues, blocking determinants that are essential for protein-protein interactions, or critical long-range effects, could also account for inhibition by mAb33 (i.e. hypotheses 2 and 3 above). Consideration of these possible mechanisms awaits a more detailed knowledge of the structure of a functional IN-DNA complex.

Finally, one striking result from our analyses deserves special comment. Because the CTD is known to be critical for substrate binding by integrase [reviewed in [42]], the finding that some of our substitutions actually increased the affinity of full-length IN for DNA was quite unexpected. These proteins, I267A and I267A/I268A, exhibit both increased DNA binding affinity (Table 2), and an absence of the initial faster dissociation phase that is characteristic of the biphasic pattern normally observed with IN. Furthermore, tighter binding was not accompanied by an increase in -2 processing but rather by an increase in non-specific DNA cleavage with these derivatives (Figure 3). A rapid association with DNA, coupled with the fast dissociation phase seen with wild type IN might be important for positioning the viral DNA ends for processing; the later, slow dissociation may represent a stable IN-DNA complex that is required for formation of the pre-integration complex. The aberrant properties of the I267A derivatives are consistent with such a model, in which the CTD normally plays a key role in aligning the viral DNA end for proper processing.

## Materials and methods

### HIV-1 IN proteins

Construction of plasmid pET28b, encoding 6XHis – HIV-1 IN – 3CS (C56S, C65S, C280S), was previously reported [26]. Using this plasmid backbone, site-directed mutagenesis (QuikChange Site-Directed Mutagenesis Kit, Stratagene) was utilized to introduce mutations encoding the amino acid substitutions F185H and/or F185K, as well as W131D and F139D, which were previously found to increase HIV-1 IN solubility [31]. The four DNA constructs specified the following substitutions: 3CS/F185H, 3CS/F185K, 3CS/F185H/W131D/F139D, and 3CS/F185K/W131D/F139D. The constructs were confirmed by DNA sequencing. Characterization of the substituted HIV-1 IN proteins confirmed that the 3CS/F185H/W131D/F139D derivative was the most soluble among them, and had properties comparable to the wild-type protein. This hexa-substituted HIV-1 IN protein was hereafter called "soluble IN," or sIN. The sIN-expressing backbone plasmid was then utilized for subsequent site-directed mutagenesis, as per the manufacturer's (Stratagene) instructions, to generate the following CTD substituted full-length sIN derivatives: F223A, R224A, Y226A, W243A, K244A, R262A, I267A, I268A, and I267A/I268A. The 6XHis-tagged proteins were expressed in *Escherichia coli *BL21(DE3) and purified as follows: cells were grown to an OD_600 _of 0.8 with shaking at 37°C, induced with 0.2 mg/ml IPTG for 3 hr, and pelleted. The cell pellets were resuspended in 20 mM HEPES-NaOH, 0.5 M NaCl, pH 7.4 containing 1 mM PMSF and 1 μg/ml each of pepstatin, leupeptin, and aprotinin. Following the addition of 1 mg/ml lysozyme to the cell sample and incubation at 25°C for 30 min, the preparation was sonicated for 100 sec on ice. The resulting lysate was subjected to centrifugation at 10,000 *g*. The supernatant fraction was then applied to a HiTrap Chelating HP column (Amersham Pharmacia Biotech) that had been loaded with Ni^2+^, as per the manufacturer's protocol. Following sample application, the column was first washed with 10 column volumes of binding buffer (20 mM HEPES-NaOH, 0.5 M NaCl, pH 7.4) and then with 5 column volumes of binding buffer containing 50 mM imidazole. The 6XHis-tagged HIV-1 IN proteins were eluted with 10 column volumes of binding buffer containing 0.5 M imidazole. Following elution from the Ni^2+ ^chelating column, the proteins were washed and concentrated using Amicon Ultra centrifugal filters with a 10 kDa cutoff (Millipore) into storage buffer (50 mM HEPES-KOH, 0.5 M KCl, pH 7.4 containing 0.1 mM EDTA, 1 μg/ml each of pepstatin, leupeptin, and aprotinin, and 40% glycerol). The samples were then frozen quickly and stored as aliquots at -80°C. The proteins were thawed on ice immediately prior to use.

### Enzyme-linked immunosorbent assay (ELISA)

The binding affinities of anti-HIV-1 IN monoclonal antibodies were determined as previously described [28]. Minor modifications to the experimental conditions are described in the relevant figure legend.

### Enzymatic activity assays

For the *in vitro *processing reactions, 1 μM of each enzyme was incubated with 1 μM ^32^P-labeled 21 base pair model viral DNA substrate for 1 hr in HEPES Buffer (pH 7.5) as described previously [2]. Processing was measured by quantifying the -2 cleavage product after exposure of the radioactive gel using a Fuji imaging system. The *in vitro *joining activity was measured by using a modified radioactive-biotin assay developed for ASV integrase [43]. The donor, comprising the same 21 base pair U5 viral DNA substrate, was labeled on the 5'-end of the strand to be processed. The target DNA comprised a 28 base pair duplex biotinylated on each 3'-end. Two μM of enzyme was incubated with 1 μM of ^32^P-labeled donor DNA in reaction buffer (25 mM HEPES, pH 7.5, 25 mM KCl, 2 mM 2-mercaptoethanol, 10 mM MnCl2) for 15 min on ice. The target DNA was then added to a final concentration of 12 μM, the mixture was incubated for an additional 15 min on ice, and then transferred to a 37°C incubator. After 2 hrs, the reaction was quenched by the addition of EDTA, and 30 μL of streptavidin-agarose beads (Invitrogen) was added to each reaction, which was incubated at room temperature for 1 hr with gentle mixing to capture the biotin-labeled products. The slurry was subjected to centrifugation and the pellet washed 4 times with wash buffer (PBS, 0.05% SDS, 1 mM EDTA). After the final wash, the pellet was resuspended in 200 μL PBS and the radioactivity bound to the beads was determined by liquid scintillation counting.

### DNA binding assay

Surface Plasmon Resonance (SPR) spectroscopy was used to measure the binding affinities of IN to DNA using a BIAcore 1000 instrument. The sequence of the three model duplex DNA substrates used correspond to those previously published by Yi *et al*. [26]. The 5'-end of one strand from each duplex was labeled with biotin to facilitate immobilization to the surface of a streptavidin-coated biosensor chip. The model substrates represent the unprocessed 5'-LTR end, the processed 5'-LTR end, and the host-target DNA; the substrates comprise the following sequences in which the conserved sub-terminal CA/GT bases in the viral LTR are underlined:

(1) U5-END (21 bp viral DNA end substrate): top strand, 5'GTGTGGAAAATCTCTAGCAGT-3'; bottom strand, BIOTIN-GCACACCTTTTAGAGATCGTCA-5'

(2) U5-END CUT (processed viral DNA end): top strand, 5'-GTGTGGAAAATCTCTAGCA-3'; bottom strand, BIOTIN-GCACACCTTTTAGAGATCGTCA-5'

(3) TARGET (non-viral DNA sequence): top strand, 5'-CCGCGATAAGCTTTAATGCGG-TAG-3'; bottom strand, BIOTIN-CGGCGCTATTCGAAATTACGCCATC-5'.

All oligonucleotides were gel-purified, heated to 94°C in HBS buffer, and cooled slowly to room temperature, allowing proper hybridization with the complementary strand prior to immobilization on the sensor chip. The surface of the streptavidin chip was primed with two injections of 10 μl of 0.035% (w/v) SDS to remove weakly bound streptavidin. Sufficient hybridized DNA was injected to give approximately 250–300 response or resonance units (RU) after washing with 10 μl of 0.035% SDS. 20 μl of biotin was then injected to block the free binding sites and to decrease non-specific binding.

The running buffer consisted of 10 mM HEPES (pH 7.5), 150 mM NaCl_2_, 1 mM DTT, 5 mM MgCl_2_, and 0.005% surfactant P20 (bufferA). All proteins were dialysed in buffered A and quantified using Bradford assay with BSA as a standard.

Sensograms were obtained for each IN derivative by injecting various concentrations of protein, ranging from 50 to 150 nM with respect to the dimer form of IN (the predominant form under these conditions), at a flow rate of 30 μL/min. The surface of the sensor chip was regenerated after each injection of protein by washing with 10 μL of 0.035% SDS, which removed only the bound protein and did not affect the amount of DNA immobilized on the surface of the chip. Data from each sensogram were analysed using the Bioevaluation program. IN shows a characteristic biphasic dissociation as reported by Yi *et al*. [26]. To obtain the kinetic rate constant for dissociation (*k*_off _or *K*_d_) and the apparent association (*k*_on _or *k*_a_), the real time data were fitted individually to generate the apparent dissociation constant (*K*_d _= *k*_off_/*k*_on_). The slower phase of the biphasic dissociation was fitted to obtain *k*_off_. Based on published data, it was assumed that the IN binds as a dimer to a single immobilized DNA end to yield a stable complex.

## Abbreviations

Abbreviations: HIV-1, human immunodeficiency virus type 1; IN, integrase; LTR, long terminal repeat; NTD, N-terminal domain; CCD, catalytic core domain; CTD, C-terminal domain; mAb, monoclonal antibody; SPR, surface plasmon resonance; ELISA, enzyme-linked immunosorbent assay.

## Competing interests

The author(s) declare that they have no competing interests.
